# Detecting Spatial-Temporal Clusters of HFMD from 2007 to 2011 in Shandong Province, China

**DOI:** 10.1371/journal.pone.0063447

**Published:** 2013-05-21

**Authors:** Yunxia Liu, Xianjun Wang, Yanxun Liu, Dapeng Sun, Shujun Ding, Bingbing Zhang, Zhaohui Du, Fuzhong Xue

**Affiliations:** 1 Department of Epidemiology and Biostatistics, School of Public Health, Shandong University, Jinan, China; 2 Shandong Center for Disease Control and Prevention, Jinan, China; Centers for Disease Control and Prevention, United States of America

## Abstract

**Background:**

Hand, foot, and mouth disease (HFMD) has caused major public health concerns worldwide, and has become one of the leading causes of children death. China is the most serious epidemic area with a total of 3,419,149 reported cases just from 2008 to 2010, and its different geographic areas might have different spatial epidemiology characteristics at different spatial-temporal scale levels. We conducted spatial and spatial-temporal epidemiology analysis to HFMD at county level in Shandong Province, China.

**Methods:**

Based on the China National Disease Surveillance Reporting and Management System, the spatial-temporal database of HFMD from 2007 to 2011 was built. The global autocorrelation statistic (Moran’s *I*) was first used to detect the spatial autocorrelation of HFMD cases in each year. Purely Spatial scan statistics combined with Space-time scan statistic were used to detect epidemic clusters.

**Results:**

The annual average incidence rate was 93.70 per 100,000 in Shandong Province. Most HFMD cases (93.94%) were aged within 0–5 years old with an average male-to-female sex ratio 1.71, and the incidence seasonal peak was between April and July. The dominant pathogen was EV71 (47.35%), and CoxA16 (26.59%). HFMD had positive spatial autocorrelation at medium spatial scale level (county level) with higher Moran’s *I* from 0.31 to 0.62 (*P*<0.001). Seven spatial-temporal clusters were detected from 2007 to 2011 in the landscape of the whole Shandong, with EV71 or CoxA16 as the dominant pathogen for most hotspots areas.

**Conclusions:**

The spatial-temporal clusters of HFMD wandered around the whole Shandong Province during 2007 to 2011, with EV71 or CoxA16 as the dominant pathogen. These findings suggested that a real-time spatial-temporal surveillance system should be established for identifying high incidence region and conducting prevention to HFMD timely.

## Introduction

Hand, foot, and mouth disease (HFMD) is a common communicable disease which usually affects children, particularly those less than 5 years old. It is characterized by a distinct clinical presentation of fever, or vesicular exanthema on their hands, feet, mouths, or buttocks. HFMD can be caused by numerous human enteroviruses (EV) [1], with *Coxsackievirus* A16 (CoxA16) and *Enterovirus* 71(EV71) the major causative agents [2]–[6]. The transmission of HFMD occurs from person to person through direct contact with saliva, faeces, vesicular fluid or respiratory droplets of an infected person and indirectly by contaminated articles. At present, there are still no available effective vaccines or drugs against HFMD human use.

Recently, numerous large-scale outbreaks of HFMD in East and Southeast Asia [7]–[11], especially deaths caused by EV71 [12], [13], have caused major public health concerns worldwide. In particular, the epidemic situation of HFMD in China is quite serious. It has become one of the leading causes of child death and a public health priority in China [14]. There were 3419,149 HFMD cases and 1,384 fatal cases during 2008–2010 from the report of China’s Health Ministry.

Several outbreaks, mainly caused by CoxA16 and/or EV71, have been reported since 2007 in China, such as Linyi in Shandong (2007) [15], Fuyang in Anhui (2008) [16], Shanghai (2009) [17], Nanchang in Jiangxi (2010) [18], etc., suggesting that the incidence of HFMD might have variability in different regions and times. Therefore, a better understanding of the spatial-temporal distribution patterns of HFMD would help to identify areas and population at high risk, and then to formulate and take appropriate regional public health intervention strategy to prevent and control the outbreak. For example, one study found that the occurrence of HFMD in Ningbo, Zhejiang Province, had an apparent seasonal distribution with a peak in June [5]. Recently, Shandong Province has been suffering from serious HFMD epidemic, the reported cases were totally up to 448,251 and ranked top 5 among 31 provinces in China during 2007 to 2011. However, the distribution of HFMD at medium spatial scale level (county level in China) is still not clear. Thus, based on the reported data of HFMD from the China Information System for Disease Control and Prevention (CISDCP, http://www.cdpc.chinacdc.cn), we conducted the spatial and space-time scan statistics analysis in Shandong Province to explore the distribution characteristics and detect spatial and spatial-temporal clusters (hotspots) of HFMD cases.

## Materials and Methods

### Data Collection

**Figure 1:**
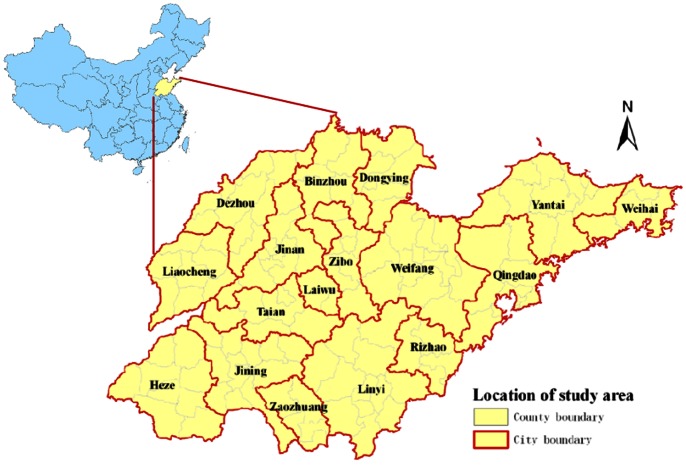
The location of study area, Shandong Province in China.

Shandong Province, located between latitude 34°25′ and 38°23′ north, and longitude 114°36′ and 112°43′ east, is a coastal province in Eastern China with a population of approximately 98 million people (Figure 1). It includes 140 counties (subdistricts) belonging to 17 regions (municipal districts) with a total land area of 156,700 square kilometers.

Data of HFMD in Shandong Province during 2007 to 2011 were derived from CISDCP, including the basic social-demographic characteristics of HFMD cases, and the pathogen type (CoxA16, EV71 and other EV) of some HFMD cases. The diagnosis was based on the clinical criteria from the HFMD Control and Prevention Guide published by the Chinese Ministry of Health [19]. The report cards of HFMD cases were filled out by professional doctors, and collected by trained reporter and then input into the CISDCP within 24 hours based on the P. R. China infectious disease prevention and cure statute. EV71, CA16 and other enteroviruses were tested by real-time PCR in 17 laboratories located in 17 cities in Shandong guided by Shandong CDC, the pathogen data was also uploaded to the China CISDCP. In this paper, we focused on the children HFMD cases (0 to 5 years) which accounted for about 94% of the total cases (described in the results section). The corresponding demographic data of each county between 2007 and 2011 were obtained from Shandong Statistical Yearbook. All collected data were geographically referenced based on 140 counties of Shandong Province, i.e., 140 spatial units for analysis.

### Statistical Analysis

The frequencies of HFMD were summarized monthly and annually by geographic area (i.e., county). The 0 to 5 years incidence rates of HFMD were calculated by HFMD counts aged 0 to 5 years divided by the population aged 0 to 5 years, which were expressed as the number of cases per 100,000. And the incidence rates of all ages were calculated using the total number of HFMD cases and total population.

The autocorrelation statistic (Moran’s *I*) [20] was used to detect the global spatial autocorrelation of HFMD cases in the study area and disclose the spatial pattern of HFMD with *Z* score at county level. The significance of Moran’s *I* was assessed by employing Monte Carlo randomization. A statistically significant (*Z* score ≥1.96) estimate of *I* indicates that neighboring counties have a similar prevalence rate of HFMD and the cases are likely to cluster at county level. The software GeoDa™ 0.9.5-i was used to conduct the analysis [21].

Scan statistics [22] were used to determine the presence of high rates clusters of HFMD. (1) Spatial scan statistic, based on discrete Poisson model, was used to identify purely spatial clusters of HFMD cases by year. The purely spatial scan statistic imposes a circular (or elliptic) window which was in turn centered on each geographical area throughout the study region. The radius of the window varies continuously in size according to the population range of the area (from zero to some upper limit specified by user). (2) Space-time scan statistic, based on Space-Time Permutation model, was adopted to determine the presence of space-time clusters of HFMD cases during the study period. Space-Time Permutation scan statistic, which is developed for the early detection of disease outbreaks and is especially useful when we have only the number of cases and no population-at-risk information [23]. This approach was defined by a cylindrical window with a circular (or elliptic) geographic base and with height corresponding to time, which undergo dynamic changes in space and time to detect possible spatial-temporal clusters [24]. The space-time scan statistic automatically adjusted for both purely spatial and purely temporal variation. The base was defined exactly as for the purely spatial scan statistic, while the height changes according to the defined time interval (less than or equal to half the total study period) which reflects the time period of potential clusters. In two scan statistics mentioned above, the null hypothesis is that the rate (Poisson model), or the independence of cases in space and time (Space-Time Permutation model), was the same within and outside the scanning window. For each window, a likelihood ratio and the relative risk were calculated to test the hypothesis. And the *P* values for detected clusters were calculated by using Monte Carlo hypothesis testing to generate a number of random replications of the data set under the appropriate null hypothesis. The scanning window with the maximum likelihood constituted most likely cluster, other windows for which the likelihood value was statistically significant were defined as secondary clusters ranked according to their likelihood ratio test statistic. The statistical analyses were done by the free SaTScan™ v9.1.1 software.

In this study, we used 140 counties of Shandong Province as spatial units, 60 months from January 2007 to December 2011 as time unit. In order to scan for small to large clusters, the largest radius was set to 50% of the total population at risk, the largest height was set to 50% of the total study period. To ensure excellent statistical power and consider the computation times, 999 Monte Carlo replications were set, and clusters with statistical significance of *P*<0.05 were all reported, including secondary clusters that did not overlap with a previously reported cluster (i.e., they had no location IDs in common). Furthermore, we used ArcGIS v9.0 (ESRI, Redlands, CA, USA) to visualize the results of scan statistic analysis.

## Results

### Prevalence of HFMD

There were 448,251 HFMD cases reported in Shandong Province from 2007 to 2011, including 9,006 severe and 79 fatal cases. The annual average incidence rate of HFMD was 93.70 per 100,000 (ranged from 35.34 per 100,000 in 2008 to 148.82 per 100,000 in 2010).

Table 1 summarized the social-demographic characteristics of HFMD cases and the pathogen types of some cases from 2007 to 2011. It showed that 0–5 year old age group was majority of the victims in the outbreaks, which accounted for 93.94% (421,072 cases) of all reported cases over the study period, with the annual average incidence rate 1442.50 (ranged from 550.11 in 2008 to 2249.54 in 2009) per 100,000. Thus, we mainly focused on 0–5 year old age group in the following spatial analysis.

**Table 1:** Social-demographic characteristics of HFMD cases and the pathogen types of some cases in Shandong Province, 2007–2011.

	2007	2008	2009	2010	2011	Total
**Age**						
0–5 year	36346	30975	131686	134181	88300	421488
>5 year	3255	2004	7469	7093	6942	26763
**Sex(0–5 year)**						
Male	23053	20000	82636	85080	55416	266185
Female	13293	10975	49050	49101	32884	155303
**Occupation (0–5 year)**						
scattered children	23808	22437	104662	98779	64398	314084
nursery children	12411	8446	26866	35286	23831	106840
other	127	92	158	116	71	564
**Pathogen (0–5 year)**						
CoxA16	–	43	38	1967	2122	4170
EV71	–	93	699	1840	3522	6154
Other EV	–	18	306	1499	1385	3208

Of 421,488 HFMD cases, 266,185 were boys and 155,303 were girls, with an average male-to-female sex ratio 1.71 (1.73 in 2007, 1.82 in 2008, 1.68 in 2009, 1.73 in 2010, and 1.69 in 2011). In China, all 0–5 year old age children spent their daytime at home, kindergarten or school. Table 1 displayed that most of HFMD cases were preschoolers (74.52% scattered children and 25.35% nursery children), the rest (0.13%) were students. From 2008, pathogen detection for HFMD had been conducting using nucleic acid testing in Shandong Province. Among 13,532 genotyped cases between 2008 and 2011, CoxA16, EV71 and other EV accounted for 30.82%, 45.48% and 23.71% respectively. Clearly, the annual distribution of HFMD pathogens varied heavily (Table 1).

**Figure 2:**
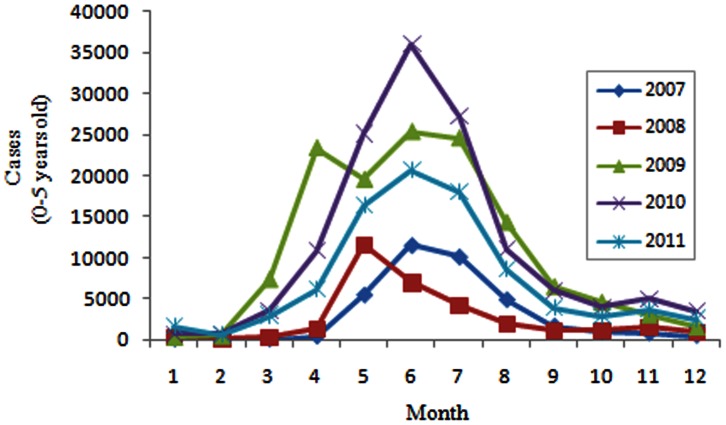
Monthly distribution of HFMD cases (0–5 years), 2007–2011.

Figure 2 illustrated the monthly distribution of HFMD cases, which indicated that the occurrence of HFMD presented significant seasonality. It was obvious that the incidence peak appeared between April and July in the study years.

### Spatial Autocorrelation of HFMD Cases

**Figure 3:**
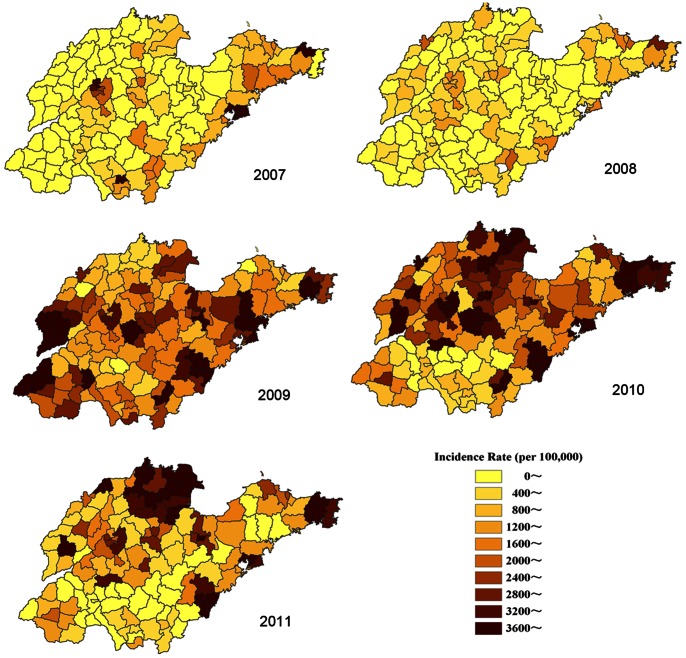
Annual incidence rates of HFMD in 0–5 years old children (/100,000) per county in Shandong Province, 2007–2011.

Figure 3 showed the annual incidence rate of 0–5 year old age per county accounting for the spatial variability of population size. It clearly indicated that the distribution of HFMD was heterogeneous at county level. Table 2 listed the results of the spatial autocorrelation test, which demonstrated that high global spatial autocorrelation of HFMD was detected at county level in Shandong Province within each epidemic year during 2007 to 2011 (Moran’s *I* >0.3, *P*<0.001).

**Table 2:** The results of the spatial autocorrelation test on HFMD cases in Shandong Province, 2007–2011.

Year	Moran’s *I*	*Z* Score	*P*-value
2007	0.6208	11.9847	<0.001
2008	0.3327	6.2828	<0.001
2009	0.3099	5.8941	<0.001
2010	0.3876	7.3657	<0.001
2011	0.4998	9.4063	<0.001

### Spatial Clusters of HFMD

**Figure 4:**
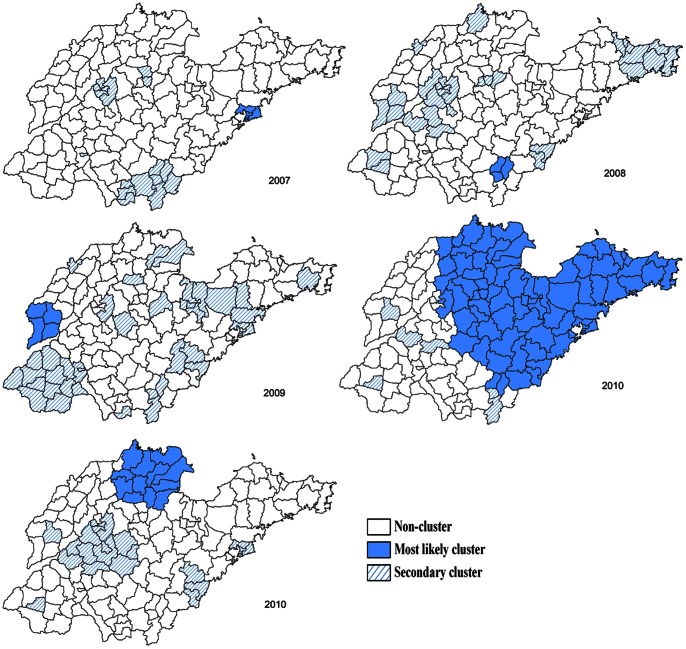
The detected purely spatial clusters of HFMD in Shandong Province, 2007–2011.

Figure 4 showed the statistically significant spatial clusters (including the most likely cluster and several secondary clusters) for a high occurrence of HFMD identified by purely Spatial scan statistic based on discrete Poisson model. It was obvious that the locations and sizes of these clusters varied by year. The most likely clusters were in Qingdao area (2007, 2010), Jinan area (2010), Linyi area (2008, 2010), Liaocheng area (2009), Zibo, Dongying and Binzhou areas (2010, 2011), Yantai, Weifang, Taian, Weihai, Rizhao, Laiwu, Dezhou areas (2011). Table 3 summarized the size, relative risk, and *P* value, etc. of most likely clusters, indicating hotspots of HFMD wandered around in the whole Shandong from 2007 to 2011.

**Table 3:** The most likely high risk clusters of HFMD cases detected using the purely spatial analysis.

Years	Cluster areas (*n*)	Radius (km)	Observed cases	Expected cases	Relative Risk	*P*-value
2007	6	14.28	5213	798.03	7.46	<0.0001
2008	3	15.77	3283	882.26	4.04	<0.0001
2009	4	32.19	12105	5297.11	2.42	<0.0001
2010	80	181.07	84702	65992.52	1.77	<0.0001
2011	12	52.83	15124	4601.58	3.76	<0.0001

### Space-time Clusters of HFMD

**Figure 5:**
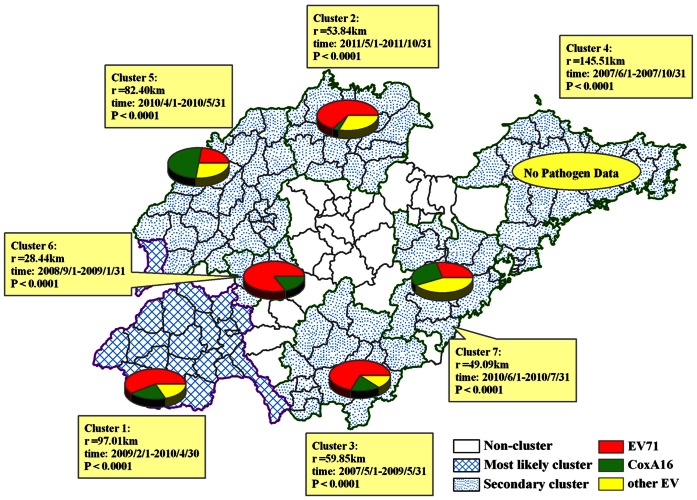
The detected spatial-temporal clusters of HFMD in Shandong Province during 2007 to 2011, and the distribution of HFMD pathogens within these clusters. Each pie shows the proportion of different HFMD pathogen for each spatial-temporal cluster, but no pathogen data in 2007.

Using Space-Time Permutation model, we detected the statistically significant monthly spatial-temporal clusters for a high occurrence of HFMD during 2007 to 2011, shown in Table 4 and Figure 5. Seven spatial-temporal clusters were detected from 2007 to 2011, including 1 most likely cluster and 6 secondary clusters. The first (most likely) cluster was located in southwest Shandong from February 2009 to April 2010 (cluster 1, Radius = 97.01 km) with 36235 cases (*RR* = 1.83, *P*<0.0001). The dominant pathogen was EV71 (57.81%). Followed by cluster in north Shandong from May 2011 to October 2011 (cluster 2, Radius = 53.84 km) with 14257 cases (*RR* = 2.46, *P*<0.0001) and EV71 as the dominant pathogen (64.37%); cluster in south Shandong from May 2007 to May 2009 (cluster 3, Radius = 59.85 km) with 23147 cases (*RR* = 1.74, *P*<0.0001) and EV71 as dominant (68.42%); cluster in northeast Shandong from June to October 2007 (cluster 4, Radius = 145.51 km) with 10488 cases (*RR* = 2.23, *P*<0.0001) and no pathogen data; cluster in northwest Shandong from June 2010 to July 2010 (cluster 5, Radius = 82.40 km) with 12097 cases (*RR* = 1.68, *P*<0.0001) and CoxA16 as dominant (50.09%); cluster in middle west Shandong from September 2008 to January 2009 (cluster 6, Radius = 28.44 km) with 1234 cases (*RR* = 6.88, *P*<0.0001) and EV71 as dominant (83.33%); cluster in middle east Shandong from June 2010 to July 2010 (cluster 7, Radius = 49.09 km) with 10180 cases (*RR* = 1.70, *P*<0.0001) and combined EV71 (29.45%) with CoxA16 (26.71%) as dominant. The above spatial pattern of clusters indicated that the occurrence of HFMD was with spatial-temporal heterogeneity. In chronological order, the earliest high occurrence area was cluster 4, followed by cluster 3, cluster 6, cluster 1, cluster 5, cluster 7 and cluster 2. Although different HFMD pathogen existed in different cluster areas, EV71 was the dominant pathogen for most clusters.

**Table 4:** The spatial-temporal high risk clusters of HFMD cases detected using Space-Time Permutation model, 2007–2011.

Clusters#	Cluster areas (*n*)	Radius(km)	Time frame	Observed cases	Expected cases	Relative Risk	*P*-value
1	19	97.01	2009/2/1 to 2010/4/30	36235	19803.50	1.83	0.0000
2	13	53.84	2011/5/1 to 2011/10/31	14257	5798.23	2.46	0.0000
3	16	59.85	2007/5/1 to2009/5/31	23147	13286.60	1.74	0.0000
4	24	145.51	2007/6/1 to 2007/10/31	10488	4710.81	2.23	0.0000
5	26	82.40	2010/4/1 to 2010/5/31	12097	7211.86	1.68	0.0000
6	4	28.44	2008/9/1 to 2009/1/31	1234	179.29	6.88	0.0000
7	11	49.09	2010/6/1 to 2010/7/31	10180	5993.14	1.70	0.0000

# :‘1’ represents ‘Most likely cluster’; ‘2–7’ represent six ‘Secondary clusters’.

## Discussion

Shandong Province has been one of the most serious HFMD epidemic areas since 2007, with annual average incidence rate 93.70 per 100,000. Most HFMD cases (93.94%) were aged less than 5 years old of both scattered children (74.52%) and nursery children (25.35%), with an average male-to-female sex ratio 1.71, and the incidence single peak season was between April and July (see Table 1 and Figure 2). The dominant pathogen was EV71 (45.48%), followed by CoxA16 (30.82%), and other EVs (23.70%). Although these epidemiological characteristics was similar to the epidemic areas in other districts of China, some different epidemic features emerged in different districts [25]. For example, though with similar pathogen pattern to Shandong Province, there were double seasonal peaks with the highest occurrence between April and June and the second occurring in November in Jiangsu Province [26]. In Hong Kong, both warmer seasonal peak (May-July) and winter peak (October-December) were detected with EV71 as dominant pathogen, but the number of older children (>5 years) infected increased from 25.4% in 2001 to 33.0% in 2009 [27]. In Guangdong Province, HFMD incidence peaked in April/May and September/October, also with EV71 (22.4%) and CoxA16 (23%) as dominant pathogen [28]. These difference might be partly attributed to climatic, geographic, social factors, etc. [28]–[30].

In Shandong Province, HFMD had positive spatial autocorrelation at medium spatial scale level (county level) with higher Moran’s *I* from 0.31 to 0.62 (P<0.001), and the high incidence areas wandered around in the whole Shandong from 2007 to 2011 (see Table 2 and Figure 3). This was similar to the spatial autocorrelation patterns in the whole mainland China at province spatial scale level [31]. Further spatial autocorrelation analysis should be done to demonstrate its spatial epidemic behavior at much smaller spatial scale level, including town and village level.

The hotspots of HFMD wandered around in the whole Shandong from 2007 to 2011 from the results of Spatial scan analysis (see Figure 4). Furthermore, using spatial-temporal analysis, 7 spatial-temporal clusters were detected, including 1 most likely cluster and 6 secondary clusters with different pathogen proportion, indicating that the occurrence of HFMD was with spatial-temporal heterogeneity (see Figure 5). Therefore, HFMD propagates in a composite space-time domain rather than showing a purely spatial and purely temporal variation in Shandong. In addition, EV71 was the dominant pathogen for most clusters, though different HFMD pathogen existed in different hotspot areas (see Figure 5). Analogously, the spatial-temporal distribution of HFMD was also nonrandom in Jiangsu Province [26] and in the landscape of whole mainland China [32]. A study had shown that this spatial heterogeneity was associated with the monthly precipitation types of the region [33]. For establishing the appropriate regional prevention strategy and measure, further study need to be done on risk factors of HFMD.

In this study, Scan statistics were used to detect the spatial and spatial-temporal clusters. The method had been widely used to detect clusters of infectious disease and may serve as a useful adjunct to disease surveillance, particularly in areas of limited resources, e.g., malaria [34], dengue [35], Japanese encephalitis [36], tuberculosis [37] and HFMD [32], etc. However, the Scan statistics are based on the assumption of circular spatial scanning windows and space-time cylinders to detect clusters, while the actual shapes of clusters were not all like that. This limitation prompts us to explore better statistic model to conduct further spatial epidemiology analysis.

In summary, this study highlighted the spatial epidemiological characteristics of HFMD at medium spatial scale level from 2007 to 2011 in Shandong Province, China. It indicated that the spatial-temporal hotspots of HFMD wandered around in whole Shandong from 2007 to 2011 with EV71 or CoxA16 as the dominant pathogen. These findings suggested that a real-time spatial-temporal surveillance system should be established for identifying high incidence region and for conducting prevention to HFMD timely.
